# Clinical implication of prognostic and predictive biomarkers for castration-resistant prostate cancer: a systematic review

**DOI:** 10.1186/s12935-020-01508-0

**Published:** 2020-08-26

**Authors:** Shengri Tian, Zhen Lei, Zuo Gong, Zhonghai Sun, Dongyuan Xu, Minhu Piao

**Affiliations:** Department of Urology, Yanbian University Hospital, Yanji, Jilin China

**Keywords:** Prostate cancer, Metastatic cancer, Biomarker, Prognostic, Predictive

## Abstract

**Background:**

Diagnosis of metastatic castrate resistant prostate cancer (mCRPC) with current biomarkers is difficult and often results in unnecessary invasive procedures as well as over-diagnosis and over-treatment. There are a number of prognostic biomarkers for CRPC, but there are no validated predictive biomarkers to guide in clinical decision-making. Specific biomarkers are needed that enable to understand the natural history and complex biology of this heterogeneous malignancy, identify early response to treatment outcomes and to identify the population of men most likely to benefit from the treatment. In this systematic review, we discuss the existing literature for the role of biomarkers in CRPC and how they aid in the prognosis, treatment selection and survival outcomes.

**Methods:**

We performed a literature search on PubMed and EMBASE databases from January 2015 through February 2020 in accordance to Preferred Reporting Items for Systematic Review and Meta-Analysis guidelines. Articles were assessed to identify relevant observational studies and randomized controlled trials regarding biomarkers which aid in identifying progression to mCRPC as well as predictive biomarkers which help in treatment selection.

**Results:**

We identified 3640 number of hits of which 58 articles were found to be relevant. Here we addressed biomarkers in the context of prognosis, prediction and patient selection of therapy. These biomarkers were found to be effective as prognostic or predictive factors under variety of conditions. The higher levels for all these biomarkers were associated with shorter median OS and sometimes PFS. Lower amounts of biomarkers in serum or urine were associated with prolonged survival outcomes, longer time to CRPC development or CRPC progression and longer median follow-up irrespective of any therapy.

**Conclusion:**

We observed that the biomarkers included in our study predicted clinically relevant survival outcomes and treatment exposure. Though the current biomarkers are prognostic when measured prior to initiating treatment, not all are validated as predictive markers in post treatment setting. A greater understanding of biomarkers in CRPC is need of the hour for development of more personalized approach to maximize benefit and minimize harm in men with CRPC.

## Background

Prostate cancer (PCa) is the second most common cancer in men and a vital cause of cancer-related morbidity and mortality globally [1]. According to International Agency for Research on Cancer, the 5-year prevalence rates in China is 30.26% in 2018 with an estimate of 99,322 new cases of PCa [2]. Patients presenting with the advanced disease typically receive hormonal therapy using medical or surgical castration as initial treatment [3]. However, most prostate cancer patients acquire resistance to the initial hormonal therapy and develop castration-resistant prostate cancer (CRPC) within 5 years from diagnosis [4].

Initially, docetaxel and hormonal manipulation were the only available strategies to manage the patients with CRPC [5]. Recently, there has been a rapid increase in the treatment options available, including novel androgen receptor-directed therapies (abiraterone acetate and enzalutamide), radiopharmaceutical (^223^radium), immunotherapeutic (sipuleucel-T), and chemotherapeutic (cabazitaxel) drugs. These drugs have shown efficacy in terms of survival outcomes in phase 3 clinical trials and consequently have been recommended in the recent treatment guidelines for CRPC [6]. Therefore, it is becoming essential to understand the optimal and rational combination and sequences of these treatments in clinical practice so as to identify patients most likely to benefit from a specific treatment. Minimizing harms and costs of ineffective therapies is another equally important goal [7].

CRPC is characterized by a heterogeneous natural history and despite the availability of these treatment options, CRPC remains a lethal disease [8]. The variable response observed in the targeted therapies could be due to the biologic heterogeneity of CRPC, including both AR-mediated or AR-independent pathways [9]. Over recent decades, the development of molecular biomarker assays and genetic assays has provided an avenue for PCa biomarker development [10]. Prognosis of patients can be estimated by prognostic models and nomograms; however, response to the therapies are not predictable. Emerging biomarkers utilize serum, urinary, or tissue samples as a test substrate [10]. In clinical practice, the utility of these biomarkers is variable and may be used at different time points throughout the care of a patient with suspected or diagnosed PCa. Specifically, these biomarkers assist in diagnosis, guiding definitive treatment options, determine the risk of ongoing monitoring versus intervention, or provide risk stratification in the setting of negative initial biopsy [10].

Prostate-specific antigen (PSA) is a widely used marker of diagnosis and prognosis; however, there is evidence of disconnection between PSA level changes and survival outcomes. Sipuleucel-T treatment extends overall survival (OS) in metastatic CRPC patients; however, it has little effect on the PSA level [11]. Whereas, bevacizumab with docetaxel did not significantly improve survival but greatly reduced PSA levels [12]. Additionally, radium-223 chloride demonstrated an OS benefit in patients with metastatic CRPC but had no clear effect on PSA levels [13]. Clinicians thus need predictive biomarkers to select treatment choices for individual patients. Similarly, prognostic biomarkers provide information about a patient’s disease outcome independent of therapy [14]. New biomarkers have been discovered owing to the recent advances in the metabolomic, genomic, and transcriptomic analysis, which can be utilized in the prediction of PCa outcome and response to therapy [15]. This systematic review was conducted to evaluate the available evidence on the prognostic and the potential predictive biomarkers in CRPC and to discuss the clinical implications of these markers on the patients.

The following questions were evaluated in completing this overall objective.

- What are the currently available prognostic biomarkers that aid in predicting clinical outcomes for progression to CRPC?
- What is the role of the predictive biomarkers in the treatment selection for CRPC patients and are they helpful in clinical decision making?

## Methods

A review protocol was developed and registered on Prospero with registration number CRD42020181860.

### Evidence acquisition

#### Search strategy

A systematic review of the literature was conducted from January 2015 to February 2020 by searching National Center for Biotechnology Center (NCBI), PubMed and EMBASE database. The following search string was used for screening of relevant literature in PubMed and EMBASE databases with minor changes in Boolean signs to suit the database: (“prostate cancer” OR “cancer of the prostate” OR “prostatic cancer” OR “castration-resistant prostate cancer” OR “non-metastatic prostate cancer” OR “hormone sensitive prostate cancer”) AND (“tumor marker” OR “biomarker” or “biologic markers” OR “serum markers” OR “surrogate marker” OR clinical marker” OR “tumor marker” OR “urine biomarkers”) AND (“survival” OR “progression free survival” OR overall survival” OR “prognostic factor/s” OR “predictive factor/s” OR “clinical outcomes”).

#### Study eligibility

Studies were selected for review based on the following criteria: (1) patients progressing from hormone sensitive prostate cancer (HSPC) or non-metastatic prostate cancer to CRPC or with mCRPC, (2) randomized clinical trials (RCTs), (3) observational studies, (4) English language, (5) Studies reporting outcomes based on prognostic and/or predictive biomarkers, (6) Patients on any therapy. Studies were excluded if they fell under the following criteria’s: (1) non-English language, (2) non-RCTs, (3) duplicate publications, (4) conference abstracts, (5) meta-analyses and systematic reviews, (6) not reporting appropriate outcomes. This review was performed in accordance to Preferred Reporting Items for Systematic Review and Meta-Analysis (PRISMA) guidelines.

Initially, titles were reviewed to assess whether they met the inclusion criteria. These studies were categorized into three categories: excluded, included and possibly relevant. Included and possibly relevant studies were rescreened to confirm eligibility.

### Evidence synthesis

Only articles that clearly defined the intended study population, with or without interventions, and clinical endpoints including progression-free survival (PFS) and overall survival (OS) (biomarker-associated, clinical or radiographic), time to follow-up, significant cut-off for being a predictive or prognostic biomarker, time to CRPC progression, time to CRPC development, were included in this review.

### Data extraction and quality assessment

Data from included studies regarding author, year of publication, title, study design, demographics of the study population and outcomes of interest was extracted by two independent reviewers into standardized MS Office Excel. The methodological quality of eligible RCTs was determined using the JADAD scale [16] and Newcastle–Ottawa scale [17] was used for observational studies.

## Results

**Fig. 1:**
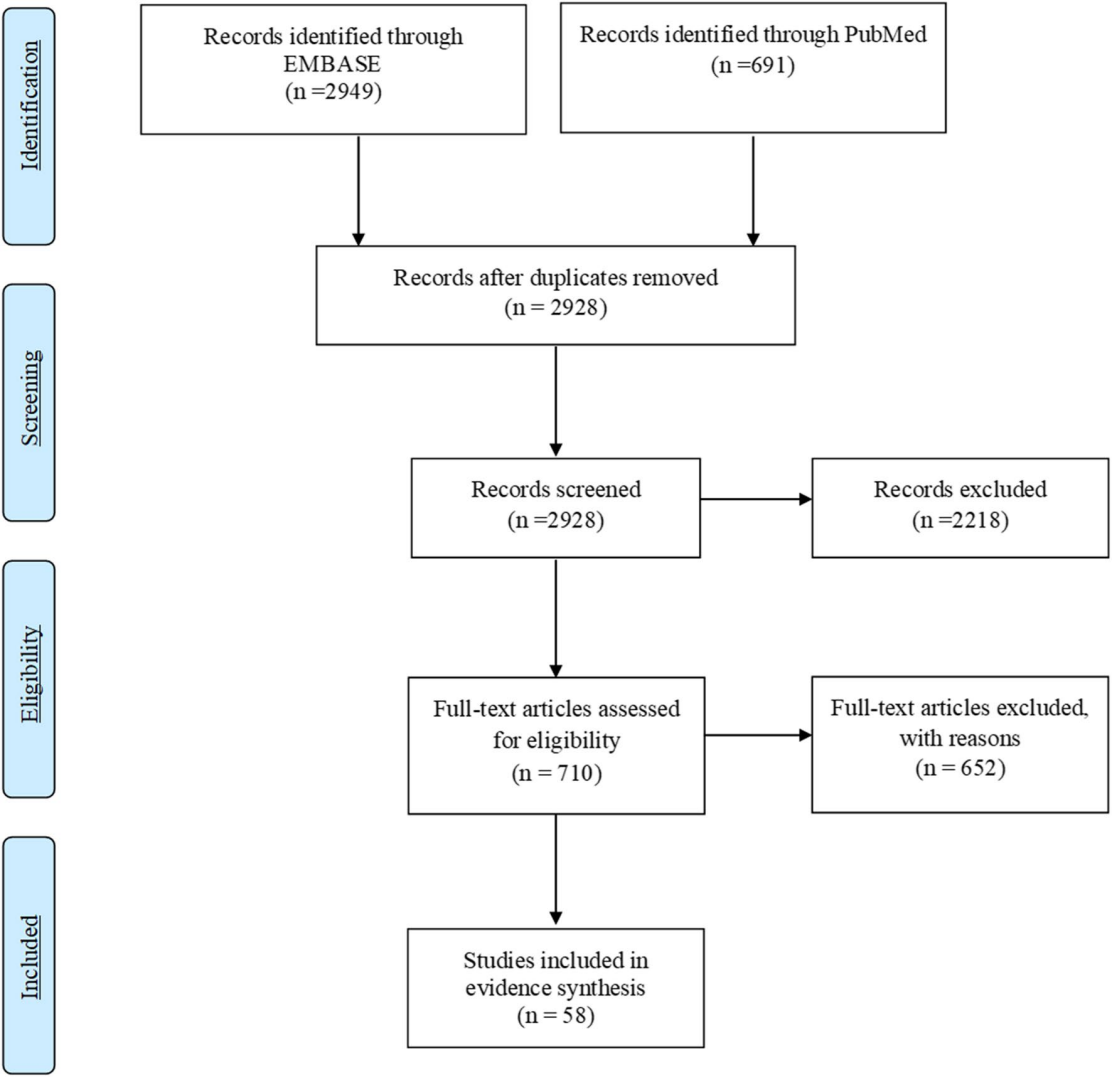
PRISMA flow diagram of the literature search process

The literature search identified in total 3640 articles. After initial title screening and manual reduplication, 712 studies were excluded (not relevant to the topic or not original research) and 2928 references remained for abstract review. Full-text evaluation for the remaining 710 citations identified by abstract review or by a manual search of the references list was done (Fig. 1). A total of 58 articles that investigated as prognostic and predictive biomarkers in development of CRPC or its progression were finally included in the study. The summary of included studies characteristics along with quality assessment is described in Table 1.

**Table 1:** Study characteristics and quality assessment of all the included studies for biomarkers

Article	Year	No of patients	Study type	Quality assessment^a^
Yasouka et al. [48]	2019	44	Observational	4
Lin et al. [53]	2018	216	Observational	6
Kosaka et al. [49]	2018	45	Observational	3
Pei et al. [51]	2019	170	Observational	4
Sathekge et al. [60]	2019	73	Observational	2
Alvim et al. [63]	2019	124	Observational	6
Armstrong et al. [70]	2018	872	Observational	4
Hamano et al. [57]	2019	321	Observational	6
Yang et al. [52]	2015	39	Observational	4
Houede et al. [65]	2015	306	Observational	4
Kuo et al. [56]	2015	62	Observational	5
Schiff et al. [64]	2019	110	Observational	3
Rahbar et al. [61]	2017	104	Observational	4
Ahmadzadehfar et al. [62]	2017	100	Observational	4
Ji et al. [54]	2017	185	Observational	4
He et al. [55]	2017	92	Observational	4
Belderbos et al. [50]	2019	224	Observational	4
Chang et al. [66]	2019	77	Observational	5
Fan et al. [67]	2018	60	Observational	7
Fukuoka et al. [58]	2019	63	Observational	4
Kodama et al. [87]	2019	575	Observational	6
Papazoglou et al. [69]	2016	44	Observational	4
Miyake et al. [68]	2017	297	Observational	4
Vaishampayan et al. [29]	2019	20	Observational	4
Dizdarevic et al. [32]	2018	57	Observational	4
Naito et al. [31]	2019	20	Observational	3
Miyoshi et al. [33]	2019	32	Observational	4
Lara et al. [30]	2018	750	RCT	4
Hammerrich et al. [34]	2017	89	Observational	5
Anand et al. [35]	2016	62	Observational	5
Onal et al. [36]	2019	102	Observational	6
Loubersac et al. [37]	2019	1082	RCT	3
Tatenuma et al. [38]	2018	73	Observational	4
Kumano et al. [39]	2019	106	Observational	4
Lorente et al. [40]	2015	755	RCT	2
Koo et al. [41]	2019	303	Observational	6
Ando et al. [44]	2019	164	Observational	5
Hashimoto et al. [45]	2019	115	Observational	6
Shiota et al. [46]	2018	106	Observational	4
Wang et al. [47]	2017	206	Observational	6
Sieuwerts et al. [18]	209	124	Observational	4
Belderbos et al. [19]	2019	127	Observational	4
Cattrini et al. [20]	2019	39	Observational	4
Qu et al. [21]	2014	250	Observational	6
Antonarakis et al. [22]	2017	202	Observational	5
Qu et al. [23]	2017	171	Observational	6
Carles et al. [24]	2018	45	Observational	5
De Kruihiff et al. [25]	2019	114	Observational	4
Bitting et al. [26]	2015	89	Observational	4
Josefsson et al. [27]	2017	53	Observational	5
Kobayashi et al. [71]	2019	104	Observational	6
Hiew et al. [72]	2018	270	Observational	3
Gravis et al. [73]	2015	385	Observational	4
Mori et al. [74]	2017	69	Observational	4
Miyoshi et al. [75]	2018	45	Observational	4
Ohtaka et al. [76]	2017	49	Observational	4
Song et al. [42]	2016	71	Observational	5
Berg et al. [43]	2015	194	Observational	5

^a^ Quality assessment of the RCTs were done using Jadad scale and non-RCTs was done using Newcastle–Ottawa scale

### Prognostic biomarkers

#### Androgen receptor (AR) splice variants in CTC

Six studies were observed for the presence of ARVs in CTC. The presence of AR-V9-positive CTCs at baseline in mCRPC was associated with poor survival outcome to cabazitaxel treatment [18], while another study reported no association of AR-V7 with OS on treatment with cabazitaxel [19]. ARV7+ was associated with shorter OS on treatment with androgen-receptor signaling inhibitors (ARSi) [20]. Further, after transurethral resection of prostate, AR-V7 expression was found to be a significant prognosticator for the development of CRPC (HR 2.627, 95% CI 1.480–4.663, p = 0.001) [21]. Similarly, ARV7+ patients had worst outcomes on OS on treatment with abiraterone acetate and enzalutamide [22, 23] (Table 2).

**Table 2:** Summary of included studies for prognostic biomarkers

Article	Year	Biomarker	Intervention	Significant outcomes
Sieuwerts et al. [18]	2019	ARV	Cabazitaxel	Median OS: 7.7 months (95% CI 7.0–10.6) Median OS (ARV7− vs ARV7+): 9 vs 3.7 months
Belderbos et al. [19]	2019	ARV	Cabazitaxel	Median OS: HR: 1.33, 95% CI 0.81–2.15, p = 0.25 Median OS (ARV7− vs ARV7+): 12.6 vs 12.3 months
Cattrini et al. [20]	2019	ARV	ARAT	Median OS: 4.7 months (95% CI 0.6–8.9)
Qu et al. [21]	2014	ARV	TURP	Time to CRPC: 9.0 months Median follow-up: 25 months Median CSS: 17 months OS (ARV7− vs ARV7+): HR (95% CI), 2.247 (1.066–4.737) 0.033
Antonarakis et al. [22]	2017	ARV	Abiraterone or enzalutamide	Median follow-up (CTC−, CTC+/AR-V7− and CTC+/AR-V7+): 15.0, 21.7, and 14.6 months Median OS (CTC−, CTC+/AR-V7− and CTC+/AR-V7+): HR (95% CI), 28.7 (28.4 to not reached, 29.5 months (18.4 to not reached), 11.2 months (8.3 to 17.1) Median follow-up: 15.0, 21.7, and 14.6 months Time to CRPC: 23.0, 20.5 and 14.0 months
Qu et al. [23]	2017	ARV	Abiraterone or enzalutamide	Median OS in abiraterone (ARV7− vs ARV7+): 35.6 vs 27.2 month Median OS in enzalutamide (ARV7− vs ARV7+): 29.1 vs 13.8 months Median TTF (abiraterone vs enzalutamide): 10.3 vs 3.7
Carles et al. [24]	2018	CTC	Radium-223	Median OS: 16 months Median OS (> 5CTC):16 months Mean follow-up: 9 ± 6 months
De Kruihiff et al. [25]	2019	CTC	Cabazitaxel	Median PFS for CTC < 5 CTC at baseline vs < 5 CTC after treatment: 8.7 months ≥ 5 CTC at baseline vs < 5 CTC after treatment: 6.4 months < 5 CTC at baseline vs ≥ 5 CTC after treatment: 7.4 months ≥ 5 CTC at baseline vs ≥ CTC after treatment: 3.5 months Median OS for CTC < 5 CTC at baseline vs < 5 CTC after treatment: 19 months ≥ 5 CTC at baseline vs < 5 CTC after treatment: 12.8 months < 5 CTC at baseline vs ≥ 5 CTC after treatment: 23 months ≥ 5 CTC at baseline vs ≥ CTC after treatment: 6.9 months
Bitting et al. [26]	2015	CTC	Abiraterone, enzalutamide	Median OS: 11.2 months Median PFS: 4.4 months Median OS (< 5 CTC vs > 5 CTC):16.6 vs 8.9 months Median PFS (< 5 CTC vs > 5 CTC): 5.7 vs 3.7 months
Josefsson et al. [27]	2017	CTC	ADT	Median PFS (CTC+ vs CTC−): 8.5 months Median follow-up: 11.1 months

*PFS* progression free survival, *OS* overall survival, *ADT* Androgen deprivation therapy

#### Number of circulating tumor cell count (CTC)

Four studies were associated with CTC as biomarker. Patients with baseline CTC counts > 5 cells/7.5 ml showed decreased OS and lower adherence to radium-223 therapy in a study [24]. Patients with < 5 CTCs prior to start of cabazitaxel therapy was prognostic indicator of better PFS and OS as compared to patients with ≥ 5 CTCs at baseline (both p < 0.001) [25]. Low CTC count was associated with longer OS than high CTC count [16.6 months (95% CI 11.7, 20.9) and 8.9 months (95% CI 6.3, 11.2)] on treatment with abiraterone or enzalutamide [26]. Similarly, CTC-positive patients were associated with shorter PFS [HR: 7.2 (95% CI 1.7–31.0; p < 0.01)]. Also, CTC-positivity (p < 0.001; HR 5.02; 95% CI 2.13–11.9) at 3 months after the start of ADT were negative prognostic markers of early progression [27] (Table 2).

### Predictive biomarkers

#### Bone turnover markers

Most of the prostate cancer patients develop significant bone pain when progressed to CRPC [28]. Seven articles assessed the predictive role of bone biomarkers in the treatment selection for CRPC. Early changes in serum/urine biomarkers (N-telopeptide-NTx and bone alkaline phosphatase-BAP) did not predict clinical benefit in mCRPC patients with cabozantinib therapy or docetaxel with/without atrasentan [29, 30]. Patients with good bone scan index response had better performance status and achieved OS prolongation when treated with radium‐223 [31]. Further, normal total alkaline phosphatase (tALP) was associated with longer OS than with elevated tALP (p = 0.01) in patients treated with ^223^Ra-Dichloride [32]. Automated bone scan index (aBSI) as a predictive marker showed no significant difference in OS from baseline to 16 weeks of treatment with cabazitaxel (p = 0.72) [33]. Patients with fast alkaline phosphatase velocity (APV) values (≥ 5.42 U/l/y) and faster PSA doubling time (PSADT) (p = 0.0289) had significantly shorter median post-CRPC BAP values (p ≤ 0.0001) with androgen deprivation therapy (ADT) [34]. The combined predictive model of percent PSA change and change in automated BSI (C-index 0.77) was significantly higher than that of percent PSA change alone (C-index 0.73), p = 0.041 in enzalutamide treated patients [35] (Table 3).

**Table 3:** Summary of included studies for predictive biomarkers

Article	Year	Biomarker	Intervention	Significant outcomes
Vaishampayan et al. [29]	2019	Bone biomarker	Cabozantinib	Median PFS: 4.1 months Median OS: 11.2 months Median change (BSAP) pre and post therapy: 21.3% Median change in serum Ntx pre and post therapy: − 13% Median change in urine Ntx pre and post therapy: − 41.7%
Dizdarevic et al. [32]	2018	Bone biomarker	^223^Ra-Dichloride	Median follow-up: 266 days ALP OS: 298 days Median OS (Normal ALP vs elevated ALP): 401 vs 222 days Median OS (ALP ≥ 30% reduction vs ALP non-responders): 363 vs 115 days Median OS (ALP ≥ 10% reduction vs ALP non-responders): 256 vs 137 days
Naito et al. [31]	2019	Bone biomarker	^223^Ra-Dichloride	Median OS: HR, 0.21; 95% CI 0.045–0.95
Miyoshi et al. [33]	2019	Bone biomarker	Cabazitaxel	Median OS: 16.2 months Median BSI level: 4.4% (range 0.1–12.9%) Median PSA level: 194.9 ng/ml (range 1.3–2611.0 ng/mL) Time to CPRC: 9.5 months Median ΔBSI: 23.5%
Lara et al. [30]	2018	Bone biomarker	Docetaxel + prednisone + atrasentan	Median OS (CICP: ≤ 6.8): 31.6 months Median OS (BAP ≤ 90.9): 27.1 months
Hammerrich et al. [34]	2017	Bone biomarker	ADT	APV ≥ 5.42 U/l/y vs APV < 5.42 U/l/y: 24.7% vs 75.3% Follow-up time (fast APV vs slow APV): 63.4 months
Anand et al. [35]	2016	Bone biomarker	Enzalutamide	Median OS: 83 weeks C-index of aBSI: 0.72 ΔBSI: median = 0.05, IQR: [−] 0.28–1.43) C-index of % of PSA change and aBSI: 0.77 Median follow-up: 56 weeks
Onal et al. [36]	2019	NLR	Abiraterone either pre- or post-chemotherapy	Median follow-up: 24 months Median OS: 20.8 months (IQR: 17.3–24.4 months) Median OS (NLR < 3.1 vs ≥ 3.1): 10.5 vs 6.5 months HR: 3.13; 95% CI 1.67–5.88; p <0.001 HR: 3.30; 95% CI 1.33–8.19; p = 0.01 NLR PFS: HR, 2.25; 95% CI 1.44–3.51; p < 0.001
Loubersac et al. [37]	2019	NLR	Abiraterone + prednisone or prednisone	Median OS (NLRlow vs NLR_high_): HR, 0.66; 95% CI 0.50–0.86, vs HR, 0.84; 95% CI 0.67–1.04 p = 0.002
Tatenuma et al. [38]	2018	NLR	Docetaxel	Median OS: 21.0 months Median OS (NLR > 2.59 vs NLR < 2.59): 12.0 vs 31.6 months
Kumano et al. [39]	2019	NLR	Enzalutamide	Median OS (NLR): HR = 4.57; 95% CI 1.31–15.96; p = 0.01 Median OS (NLR > 14 vs < 14): 17.9 months vs 22.0 months
Lorente et al. [40]	2015	NLR	cabazitaxel versus mitoxantrone	Median OS: 14 months (95% CI 13.2–14.8) BLNLR > 3 vs < 3 on PSA response: 40.1% vs 59.9% Median follow-up: 12.8 months
Koo et al. [41]	2019	NLR		Median follow-up: 18.5 months Median RFS:3.7 (2.3–8.3) OS (NLR < 2.5 vs > 2.5): 23.5% vs 14.5%)
Miyoshi et al. [75]	2018	ERG	ADT	Median time to CRPC: 40.2 months Median time to CRPC with PTP (high vs low):14.8 months vs 86.3 months
Ohtaka et al. [76]	2017	ERG	ADT	Median overall OS high PTP: Not reached Median overall OS low PTP: 23.8 months

*PFS* progression free survival, *OS* overall survival, *ADT* Androgen deprivation therapy

#### Neutrophil lymphocyte ratio

Six studies were analysed for the role of NLR as biomarker. High-NLR (≥ 3.1) patients predicted worse OS and PFS in patients treated with abiraterone acetate than low NLR patients [36, 37]. Similar observations were noted in another two studies in patients with NLR_low_ and on docetaxel when NLR cut-off was 2.59 and 2.14 [38, 39]. Treatment of cabazitaxel over mitoxantrone was favored due to demonstration of higher median OS [15.9 vs 12.6 months, HR 1.55 (95% CI 1.3–1.84), p < 0.001], PSA progression-free survival [3 vs 3.1 months; HR 1.35 (95% CI 1.12–1.62); p = 0.002] and radiographic progression-free survival [9.3 vs 5.7 months; HR 1.42 (95% CI 1.15–1.76); p = 0.001] in patients with NLR cut-off < 3 than with NLR ≥ 3 [40]. Further another study reported that NLR ≥ 2.5 was an independent predictor of a lower risk for CSS in patients treated with docetaxel [41] (Table 3).

#### ERG

Only two articles were available for screening of ERG as biomarker. ERG positivity correlated with a lower PSA-PFS (3.2 months vs 7.4 months, *p *< 0.001), C/R-PFS (3.8 mos vs 9.0 mos, *p *< 0.001) and OS (10.8 mos vs 21.4 mos, *p *< 0.001), thus indicating that ERG is potential biomarker for prediction to docetaxel treatment in mCRPC patients [42]. However, another study showed that ERG expression was not associated with risk of CRPC for predicting response to primary ADT in mCRPC patients [43] (Table 3).

### Predictive/prognostic biomarker

#### Testosterone

Role of testosterone as biomarker was assessed in four included studies. Testosterone ≥ 13 ng/dl was an independent prognostic factor of OS and PFS for patients treated with docetaxel. The high‐testosterone (TST) group had significantly shorter OS and PFS than the low‐TST group. Furthermore, a high serum TST predicted poor post‐docetaxel survival in patients who received subsequent therapy, including ARAT and/or cabazitaxel [44]. A serum testosterone level of 5 to < 50 ng/dl was a significant predictor for determining the efficacy of AR-targeted therapy [45]. PFS and OS when serum testosterone level was > 0.05 ng/ml in patients treated with ADT was significantly superior than with testosterone level below 0.05 ng/ml [46]. Testosterone levels of ≤ 25 ng dl/1 after the first month of ADT offered best overall sensitivity and specificity for prediction of a longer time to CRPC (p = 0.013) and was significantly associated with a lower risk of progression to CRPC (adjusted HR, 1.46; 95% CI 1.08–1.96; p = 0.013). The result showed that time to CRPC was related to testosterone levels (p = 0.020) [47] (Table 4).

**Table 4:** Summary of included studies for predictive/prognostic biomarkers

Article	Year	Biomarker	Intervention	Significant outcomes
Ando et al. [44]	2019	Testosterone	Docetaxel	Median OS: 35.8 months Median OS (TST > 13 ng/dl vs < 13 ng/dl): 19.2 vs 76.9 months Median PFS (TST > 13 ng/dl vs < 13 ng/dl): 5.1 vs 7.1 months Median follow-up: 21.6 months
Hashimoto et al. [45]	2019	Testosterone	Abiraterone or enzalutamide	Median follow-up: 26 months Median PFS (< 5 ng/dl vs 5 ng/dl): 12.2 vs 4.5 months
Shiota et al. [46]	2018	Testosterone	Enzalutamide, abiraterone, docetaxel, cabazitaxel	PFS (T < 0.05 vs > 0.05): p = 0.047 OS (T < 0.05 vs > 0.05): p = 0.18
Wang et al. [47]	2017	Testosterone	ADT	Median time to CRPC (T < 25 ng/dl vs > 25 ng/dl): 19.1 vs 14.6 months Median follow-up: 14 months
Yasouka et al. [48]	2019	PSA	Cabazitaxel	Median follow-up: 13.2 (IQR) = 6.9–21.5 months 45.5% Median PFS: 4.3 months Median OS: 20.7 months PSA (> 100 ng/ml):HR = 3.65, 95% CI 1.39–10.60, p = 0.0085
Lin et al. [53]	2018	PSA	ADT	nPSA > 0.2 ng/ml: HR, 2.665, 95% CI 1.495–4.750, p < 0.001 Median follow-up: HR: 0.262, 95% CI 0.161–0.426 Median PFS: 14.0 months Median PSA: 14.7 months Median TTN: 8.10 months
Kosaka et al. [49]	2018	PSA	Cabazitaxel	Median OS: 16.1 months PSA ≥ 100 ng/ml prior to cabazitaxel: HR = 4.375; 95% CI 1.755–10.91, p = 0.002
Pei et al. [51]	2019	PSA	Docetaxel	TTN ≥ 15 weeks: HR 0.093, 95% CI 0.044–0.188, p < 0.001 PSA nadir < 4.55 ng/ml: HR 4.002, 95% CI 1.890–8.856, p = 0.001 PSA decline > 50%: HR 0.573, 95% CI 0.428–0.756, p < 0.001
Sathekge et al. [60]	2019	PSA	^225^Ac-PSMA-617	Median OS: 18 months Median PFS: 15.2 months PSA decline > 50%: p < 0.001 Median follow-up: 9 months
Alvim et al. [63]	2019	PSA	Abiraterone acetate	Median OS (PSAr): HR: 0.19; 95% CI 0.10–0.38; p < 0.001 Median PFS (PSAr): HR: 0.24; 95% CI 0.14–0.41; p < 0.001 Median OS (PSA): 11.5 months 29.3 vs 9.7 17 vs 5.3
Armstrong et al. [70]	2018	PSA	Enzalutamide	Median OS: 23.1 months Median time to PSA (no-decline or decline < 30% group): 3.7 month Median time to PSA progression: 13.8 months (95% CI 11.3–14.0)
Hamano et al. [57]	2019	PSA	Docetaxel, AA and ENZ	PSA nadir > 0.64 ng/ml and TTN < 7 months: HR, 3.34; 95% CI 1.99–5.61; p < 0.001 Median OS: (PSA nadir > 0.64 ng/ml and TTN < 7 months): HR: 2.98; 95% CI 1.77–5.02; p < 0.001 Median follow-up: 35 months
Yang et al. [52]	2015	PSA	Docetaxel	Median OS: 13.51 months Median TTN: 5.14 months
Houede et al. [65]	2015	PSA	Abiracetone acetate	PSA response > 3 months: p = 0.00025 Median OS: 14.6 months Follow-up: 36.3 months
Kuo et al. [56]	2015	PSA	ADT	Median time to PSA rise: 4.5 months Median time to PSA rises after first T > 50 ng/dl: 1.0 months Median times from primary treatment to CRPC: 9.7 years
Schiff et al. [64]	2019	PSA	Abiraterone	≥ 30% PSA at 4, 8, 12 weeks OS: range: 35.2 months to 40.0 months ≥ 50% PSA at 4, 8, 12 weeks OS: range: 37.3 months to 41.1 months
Rahbar et al. [61]	2017	PSA	^177^Lu-PSMA-617	Median OS: 56.0 weeks Median OS (PSA decline > 50% vs < 50%): 66 weeks vs 47 weeks
Ahmadzadehfar et al. [62]	2017	PSA	^177^Lu-PSMA-617	PSA decline ≥ 14 OS vs < 14: 88 weeks vs 29 weeks PSA decline ≥ 50% vs < 50%: HR: 70; 95% CI 39.5–100.5 vs HR: 49; 95% CI 30.2–67.8 Time to CRPC progression: 38 months
Ji et al. [54]	2017	PSA	ADT	PSA nadir: HR 1.185, 95% CI 1.080–1.301, p = 0.001 Velocity of PSA decline > 11 ng/ml/month: HR 2.124, 95% CI 1.195–3.750, p = 0.001 Time to PSA nadir: 9 months Median time to progression to CRPC: 38 months
He et al. [55]	2017	PSA	ADT	Mean time to CRPC: 23 months Time to reach minimal PSA (> 1-year vs < 1 year): 8.5 months vs 3.9 months
Belderbos et al. [50]	2019	PSA	Cabazitaxel	Median OS: 13.3 months Haemoglobin: OR 1.48, 95% CI 1.05–2.07, p = 0.024 Lower AP: OR 0.61, 95% CI 0.39–0.96, p = 0.034
Chang et al. [66]	2019	PSA	Abiraterone, enzalutamide	Median follow-up (AA vs Enza): 18.2 vs 14.5 months Median PFS: 7.3 months vs 9.5 months PSA nadir: HR = 1.000, 95% CI 1.000–1.001, p = 0.010 Median time to CRPC (AA vs Enza): 31.5 vs 24.9 months
Fan et al. [67]	2018	PSA	Abiraterone + prednisone vs prednisone	Median follow-up: 14 months (range 7.0–18.5 months Median PSA PFS:10.3 vs 3.0 months Median PSA rPFS: 13.9 vs 3.9 months Median OS: 23.3 vs 17.5 months Time to castration resistance < 18 months: HR, 12.8, 95% CI 2.0–83.1, p = 0.007
Fukuoka et al. [58]	2019	PSA	FGA therapy	Time to CRPC p = 0.007 Median PSA PFS: HR: 2.39, p = 0.020 Median PSA nadir > 1 ng/ml: HR: 2.40, p = 0.034 Time from starting PADT to PSA nadir ≤ 1 year: HR: 1.85. p = 0.047
Kodama et al. [87]	2019	PSA	ADT	Median follow-up: 31 months Median time to CRPC: 13 months CRPC survival (PSA < 100 vs > 100): 31 vs 18 months, Median OS (PSA < 100 vs > 100): 85 vs 78 months, p = 0.509
Papazoglou et al. [69]	2016	PSA	Enzalutamide	Median survival time from diagnosis of CRPC: 41.1 months Median PFS: 3.0 months Median OS: 6.3 months
Miyake et al. [68]	2017	PSA	Enzalutamide, abiraterone	Median time to PSA progression (TTN < 19 weeks vs TTN > 19 weeks) in Abiraterone acetate: 8.4 vs 11.1 months Median time to PSA progression (< 14 weeks vs > 14 weeks) in Enzalutamide: 11 vs 9.9 weeks
Kobayashi et al. [71]	2019	LDH/ALP	ADT	Median follow-up: 48.1 months Median PFS: 24 months Median OS: 67.4 months LDH PFS: HR: 1.42; 95% CI 1.15–1.74; p = 0.0004 LDH OS: HR = 1.46, 95% CI 1.13–1.82; p = 0.0014 ALP OS: HR = 1.04; 95% CI 1.00–1.07; p = 0.015
Hiew et al. [72]	2018	LDH	Docetaxel	Serum LDH > 450 U/l: SD:0.054; 95% CI 0.650–0.864, p < 0.001 LDH PFS: HR: 1.876, 95% CI 1.289–2.7300 LDH OS: HR: 1.630, 95% CI 1.127–2.357
Gravis et al. [73]	2015	ALP	ADT	ALP OS: 62.1 vs 23.2% ALP C-index: 0.64 95% CI 0.52–0.66 Median follow-up: 58.3 months
Mori et al. [74]	2017	LDH	Abiracetone, enzalutamide	LDH (< 210 IU/l: 17 months) vs LDH ≥ 210 IU/l: 8 months PFS: HR: 0.39 (0.15–1.03) 0.056 OS: HR: 0.79 (0.31–2.02) 0.63
Song et al. [42]	2016	Tyrosine Phosphatase	Docetaxel	PSA response (ERG+ vs ERG−): 15.4% vs 62.1%, p = 0.004 OS (ERG+ vs ERG−): 10.8 months vs 21.4 months, p < 0.001 C/R PFS (ERG+ vs ERG−): 3.8 months vs 9.0 months, p < 0.001 Mean follow-up: 52.9 ± 27.2 months
Berg et al. [43]	2015	Tyrosine Phosphatase	ADT	Median follow-up: 6.8 years (IQR: 4.9–7.3) Median time to CRPC (ERG+ vs ERG−): 3.9 years vs 4.5 years Median OS: 5.6 months

*PFS* progression free survival, *OS* overall survival, *ADT* Androgen deprivation therapy

#### PSA and PSA kinetics

About 23 included studies demonstrated the potency of PSA as biomarker on treatment with various therapies for prostate cancer. PSA > 100 ng/ml was found to be significant predictor for shorter OS in two studies [48–50] while PSA decline of > 50 or > 30% was observed to be significant in another study [49]. Higher hemoglobin level before treatment with cabazitaxel (p = 0.024) and a lower alkaline phosphatase (AP) level at the start of treatment (p = 0.034) resulted in a higher chance of PSA response in another study [50]. Time to PSA nadir (TTPN) ≥ 15 weeks was a prognostic factor associated with longer OS and PFS compared to those with a TTPN < 15 weeks (43 vs 15 months, *p* < 0.001; 24 vs 6 months, *p* < 0.001, respectively) for patients treated with docetaxel. Further, PSA nadir (nPSA) < 4.55 ng/ml were associated with longer OS and PFS (HR 4.002, 95% CI 1.890–8.856, p = 0.001) [51]. PSA response was a significant factor for longer OS and cancer-specific survival (CSS) (p = 0.014 and p = 0.05, respectively) in post-docetaxel treated patients [52].

Higher PSA nadir, higher TTN and a shorter time to PSA nadir were significant predictors of an increased risk of progression to CRPC during initial ADT and was associated with shorter PFS in ADT treated patients [53–55]. Correlation of testosterone to PSA levels during treatment with ADT showed median time to PSA rise was 4.5 months and especially after T > 50 ng/dl was a significant prognosticator associated with a 71% reduction in the risk of developing CRPC (p = 0.05) [56]. Similar observations were noted when nPSA cut-off was > 0.64 ng/ml in patients treated with ADT [57]. Time to CRPC (p = 0.007, HR = 4.77), regional lymph node involvement at the diagnosis of CRPC (p = 0.022, HR = 2.42), and PSA-PFS of alternative first generation androgen (FGA) therapy ≤ 6 months were identified as prognostic factors, while nPSA > 1 ng/ml during and time from starting FGA to nPSA ≤ 1 year were predictive factors for worse PSA-PFS in alternative FGA therapy [58]. CRPC-free survival was significantly shorter in the PSA ≥ 100 group than in PSA < 100 group in patients treated with ADT. However, the OS after CRPC diagnosis was significantly shorter in the PSA < 100 group indicating it might be a poor prognostic factor in CRPC patients [59]. PSA decline of > 50% proved significantly associated with better OS (20.1 months vs 10.5) and PFS (17.9 months vs 6.6 months) following treatment with ^225^Ac-PSMA-617 over PSA decline < 50% [60]. PSA decline ≥ 20.87% and ≥ 14% was a prognosticating indicator for longer survival, in another two studies [61, 62].

Treatment with abiraterone acetate demonstrated that PSA reduction > 30% or ≥ 50% remained predictive of better PFS and OS [63, 64]. Duration of treatment > 3 months by abiraterone acetate was significantly predictor (p = 0.00025) of treatment [65]. To determine the suitability of treatment approach, PSA response rate at > 50% and > 90% was evaluated which showed no statistically significant difference in patients treated with abiraterone acetate or enzalutamide. However, overall, nPSA (HR = 1.000, 95% CI 1.000–1.001, p = 0.010) was an independent prognostic factor for OS [66]. Time from therapy to castration resistance of ≤ 18 months was a determinant of shorter OS in another study (p = 0.007) [67]. TTPN > 19 weeks was superior to TTPN ≤ 19 weeks in abiraterone acetate group than in enzalutamide group (11.1. months vs 8.4 months) [68]. PSA response of ≥ 50% had significantly longer times to PSA progression, rPFS, and OS in patients treated with enzalutamide [69, 70] (Table 4).

#### Lactate dehydrogenase and alkaline phosphatase

Four studies assessed lactate dehydrogenase (LDH) and alkaline phosphatase (ALP) as biomarker. Serum LDH value was significantly prognostic marker for PFS (HR = 1.42, 95% CI 1.15–1.74; p = 0.00040) and OS (HR = 1.46, 95% CI 1.13–1.82; p = 0.0014), in addition to alkaline phosphatase levels for OS (HR = 1.04; 95% CI 1.00–1.07; p = 0.015) [71]. Pretreatment serum LDH was a strongest biomarker at the point of initiation of docetaxel therapy with LDH ≥ 450 U/l levels associated with poorer PFS (p < 0.00) and OS (p = 0.011). However, pretreatment serum LDH did not predict a positive response to docetaxel [72]. ALP was a strongest prognostic factor in discriminating patients with good or poor prognosis with median OS in patients with normal and abnormal ALP of 69.1 and 33.6 months treated with ADT with/without docetaxel [73]. Similarly, abiraterone acetate-enzalutamide group showed significantly longer total PSA PFS than enzalutamide–abiraterone acetate group (p = 0.049). Survival analysis showed that combined PFS was significantly longer among patients with LDH < 210 IU/l before the first ARAT than in those with ≥ LDH 210 IU/l [74] (Table 4).

#### Tyrosine phosphatase

Of the two articles included, one study reported that median time to CRPC was significantly shorter in the high tyrosine phosphatase (PTP) group (14.8 months) than that in low PTP group (86.3 months, *p *< 0.01). Thus, high PTP expression was a significant predictor of time to CRPC treated with ADT. [75] This was similar to another study by Ohtaka 2017, where PTP expression (high vs low; HR = 2.7, 95% CI 1.0–7.2, *p *= 0.04) was independent prognostic factor for OS [76] (Table 4).

## Discussion

With the growing number of various therapeutic options that can extend survival in mCRPC patients, there is a need for the biomarkers to guide in simultaneous decisions for optimal treatment and predict which patients will benefit the most from the treatment. It is unlikely that a single biomarker will provide all information we need to tell how aggressive a newly diagnosed cancer is. No immunochemical, or genetic marker is currently used to differentiate between various stages of prostate cancers. PSA is the most widely used biomarker till now preferred for screening as well in follow-up after treatment [77]. However, PSA level change is variably dependent on the mechanism of action of different treatments. For example, early declines in PSA may be observed in novel hormonal therapies such as AA or enzalutamide which are highly prognostic in nature and associated with their mechanism of action [78]. A rise in PSA while a patient is receiving androgen deprivation therapy potentially signals a transition from hormone-sensitive prostate cancer to CRPC. However, castration levels of serum testosterone must be demonstrated before castration resistance is confirmed [79]. In the above included studies, it was observed that the PSA > 100 ng/ml, nPSA > 0.2 ng/ml, a velocity of PSA decline > 11 ng/ml per month were associated with shorter OS and PFS in patients with mCRPC while PSA decline > 50% or > 30% was associated with longer survival outcomes irrespective of any therapy. Similarly, TTPN > 6 weeks were a significant prognostic factor for survival. Early PSA response > 30% or > 50% after initiation of treatment is a significant predictor for longer OS. However, PSA levels have several restrictions as a biomarker in monitoring CRPC especially in the context of novel non-cytotoxic treatments that may have little effect on its levels. Further, PSA levels may not provide accurate information regarding the extent of bone metastasis or bone-specific effects of treatment, indicating the need of alternative biomarkers for this purpose. Bone is a common site of metastases affecting more than 90% of mean at autopsy. Even though the impact of these biomarkers is not known, they provide useful information related to the survival and progression of CRPC [80]. The elevated baseline level of BAP may be predictive for survival benefit with radium-223 treatments, and post treatment BAP reductions are highly associated with improvements in survival with radium-223 chloride [13]. In our studies, elevated BAP showed poorer outcomes on survival on treatment with radium-223, while faster APV and shorter PSADT were significant predictors of poorer bone metastasis free survival and OS.

It was noted that higher NLR values (> 2.14, > 2.5 or > 3.1) predicted worse OS and CSS in patients treated with novel hormonal therapies and docetaxel chemotherapy. This was in consistence to the other studies where high NLR was associated with poorer PFS in patients with metastatic CRPC across different treatments including abiraterone, docetaxel [81, 82]. Though the biology behind higher NLR to be significant predictor is unclear, it is presumed that the increased NLR may arise from altered tumor-inflammatory cell reactions, which is an indicator of progressive malignancy [83]. Testosterone as prognostic factor demonstrated that lower TST levels were associated with significant longer time to survival to treatment with docetaxel, ARAT and ADT. The mechanism of TST exhibiting benefits at lower levels may be related to acquired resistance than primary resistance, however this role is still unclear [45]. However, one study reported that high levels (> 0.05 ng/ml) was significant predictor of OS on treatment with ARAT, thus though TST is a significant prognostic factor, the role of TST in ARAT is unclear [46].

An increased LDH level after treatment may be predictive for poor treatment response [84]. This was also observed when the LDH level was > 450 U/l in patients initiated with docetaxel [72] and > 210 U/l in patients treated with ARAT and predicted poorer OS [74]. Also serum ALP is a significant biomarker for prediction to longer OS [71, 73]. ARV is an important prognostic factor in the progression from prostate cancer to mCRPC. Higher expression of ARV in CTC and not prostate tissues is poor prognostic factor [85]. Presence of ARV7+ and CTC+ in patients with mCRPC were associated with poorer outcomes on OS along with higher ARV7 values. However, one study reported no significant association with ARV7 [18]. Circulating tumor cells (CTCs) have emerged as a viable solution to the problem whereby patients with a variety of solid tumors, including PC, often do not have recent tumor tissue available for analysis [86]. CTC count < 5 has been a good prognostic factor for the PFS and OS in patients initiated with cabazitaxel and radium-223 therapy. Presence of CTC in patients after 3 months of initiation of ADT therapy was associated as a negative marker for early progression to CRPC [27]. Lesser explored biomarkers such as tyrosine phosphatase showed that higher levels predicted poorer OS and CSS in the two included studies [75, 76] while patients with ERG positive values showed poorer outcomes of OS and PFS [42, 43].

Thus, through our review, we have given an insight on how the biomarkers are significant in determining treatment selection. A meaningful observation from our included studies was that higher levels with any of the biomarkers in urine or blood were prognostic indicator for poorer survival outcomes, early development of CRPC and shorter follow-up duration to treatment. Also, the appropriate cut-off levels for biomarker was a significant predictor for exposure to treatment in the included studies. We thus highlight the need to establish the cut-off level for particular biomarker which will be helpful for the clinicians in diagnosis of CRPC and providing a suitable treatment strategy.

## Conclusion

Diagnosis of CRPC and its management requires an individualized approach to both patient care and trial design. Although we have given a meaningful insight into the utility of the biomarkers for treatment responses and survival outcome, future research is needed with respect to the prediction of biomarker response in sequential therapy so as to design a series of optimal treatment in patients with CRPC. Currently all biomarkers in clinical use have prognostic implications when measure prior to initiating treatment, however not all are validated as predictive markers in post treatment setting. ARV7 splice variant and CTC also look like promising candidates in development of biomarkers and may benefit a specific group of CRPC population. More prospective studies on CRPC biomarkers are required to identify the surrogate value of these biomarkers on survival which will be helpful in clinical decision making.

## Abbreviations

aBSI: Automated bone scan index
ADT: Androgen deprivation therapy
ALP: Alkaline phosphatase
APV: Alkaline phosphatase velocity
BAP: Bone alkaline phosphatase
CRPC: Castration-resistant prostate cancer
CTC: Circulating tumor cell count
HSPC: Hormone sensitive prostate cancer
LDH: Lactate dehydrogenase
mCRPC: Metastatic castrate resistant prostate cancer
NCBI: National Center for Biotechnology Center
OS: Overall survival
PCa: Prostate cancer
PFS: Progression-free survival
PSA: Prostate-specific antigen
PSADT: PSA doubling time
PTP: Tyrosine phosphatase
RCTs: Randomized clinical trials
tALP: Total alkaline phosphatase
TST: Testosterone
